# Detectability of Colon Polyp Using Computed Virtual Chromoendoscopy with Flexible Spectral Imaging Color Enhancement

**DOI:** 10.1155/2012/596303

**Published:** 2012-03-05

**Authors:** Shinsuke Kiriyama, Takahisa Matsuda, Takeshi Nakajima, Taku Sakamoto, Yutaka Saito, Hiroyuki Kuwano

**Affiliations:** ^1^Department of Surgery, Gunma Chuo General Hospital, Maebashi, Gunma 371-0025, Japan; ^2^Department of General Surgical Science, Graduate School of Medicine, Gunma University, Maebashi, Gunma 371-8511, Japan; ^3^Endoscopy Division, National Cancer Center Hospital, Tokyo 104-0045, Japan

## Abstract

The aim of this pilot study was to assess the feasibility of using computed virtual chromoendoscopy with the flexible spectral imaging color enhancement (FICE) for colon neoplasia screening. A modified back-to-back colonoscopy using FICE and white light in the right-sided colon was conducted prospectively for the consecutive patients attending for the postoperative (sigmoidectomy or anterior resection) follow-up colonoscopy. Histopathology of detected lesions was confirmed by evaluation of endoscopic resection or biopsy specimens. One-hundred and two patients were enrolled, and 100 patients (61 males and mean age 63 years) were finally analyzed. The total number of polyps detected by FICE and white light colonoscopy was 65 and 45, respectively. The miss rate for all polyps with FICE (24%) was significantly less than that with white light (46%) (*P* = 0.03). Colonoscopy using FICE could beneficially enhance the detection of neoplastic lesions in the right-sided colon compared to white light colonoscopy.

## 1. Introduction

Colonoscopy is the accepted gold standard for the detection of colorectal lesions including colorectal cancers and adenomas. Early detection and removal of colorectal adenomas have been shown to be the most effective way of colorectal cancer prevention, however, polyps can be missed with conventional white light (WL) colonoscopy [1, 2]. Unfortunately, at standard WL colonoscopy, classification of lesions is often difficult and a substantial percentage of adenomas are missed during the procedure. According to the results of back-to-back colonoscopies by Rex et al., the miss rate for adenomas ≥1 cm was 6%, for adenomas 6–9 mm was 13%, and for adenomas ≤5 mm was 27%, respectively [3]. Furthermore, there was a trend toward right-sided colorectal adenomas being missed more often than left-sided ones (27% versus 21%). As missing adenomas or cancers during colonoscopy would result in increasing the need of surgery and death from colorectal cancers, attempts to reduce this kind of miss rate include pancolonic dye spraying, wide angle colonoscopy, or cap-fitted colonoscopy [4–8].

On the other hand, computed virtual chromoendoscopy with the flexible spectral imaging color enhancement (FICE) has been developed as a new dye-less imaging technique, which might allow higher rate of colon polyp detection [9–13]. FICE is based on a computed spectral estimation technology that arithmetically processes the reflected photons to reconstitute virtual images for a choice of different wavelengths. Due to its variable setting functions, it is possible to select flexibly the most suitable wavelengths required for examination. Based on technical considerations, it is conceivable that advanced virtual imaging techniques might highlight adenomas during colonoscopy, however, its effectiveness, measured as frequency of detection of colorectal polyps in comparison to conventional WL colonoscopy, has not been investigated enough. We therefore conducted this pilot study to assess the feasibility of using FICE for colon neoplastic lesions screening.

## 2. Methods

### 2.1. Study Design

From August 2008 to March 2009 in National Cancer Center Hospital, Japan, a modified back-to-back colonoscopy using FICE and WL was conducted for 102 patients in the right-sided colon including cecum, ascending and transverse colon. This study was conducted prospectively, and written informed consent for examination and treatment was obtained from all of the studied patients prior to the procedures. The consecutive patients attending for the postoperative (sigmoidectomy or rectal anterior resection) follow-up colonoscopy were randomized to undergo the colonoscopy with either FICE or WL (group A: WL-FICE, group B: FICE-WL). After randomization, the scope was inserted into the cecum using white light. Patients with known inflammatory bowel disease, overt bleeding, and polyposis syndrome and patients receiving anticoagulant medication were excluded from the study.

### 2.2. Flexible Spectral Imaging Color Enhancement (FICE)

All examinations were performed with high-resolution zoom endoscopes (EC 590 ZW, Fujifilm medical, Tokyo, Japan). However, the zoom function of the device was not utilized for this study. The system was equipped with the EPX 4400 processor (Fujifilm medical) that provides the FICE technology.

Based on preliminary experience of the participating endoscopists, FICE set 7 (R 540 nm, G 490 nm and B 420 nm) was favored over other FICE sets for application in the colon and was therefore exclusively used in this study. In the FICE turn, withdrawal was performed with activated FICE set 7. Switching back to conventional imaging was allowed at the discretion of the endoscopist only for polypectomies.

### 2.3. Endoscopic Procedure

All patients were prepared for colonoscopy by ingesting 2-3 liters of polyethylene glycolelectrolyte solution on the same-day morning. Scopolamine butylbromide (10 mg) was administered intravenously to avoid bowel movement prior to examination for the patients with no contraindication to the use of this agent. Basically all colonoscopies were performed without sedation, by one of three experienced colonoscopists (more than 1000 colonoscopies). Only when patients felt abdominal pain, midazolam (2 mg) was administered intravenously during procedure. Quality of bowel preparation was assessed by the examiner as follows: (a) excellent (near 100% mucosal visualization following suction of fluid residue), (b) good (near 90% mucosal visualization), and (c) fair (less than 90% mucosal visualization). Examinations were performed in a modified back-to-back fashion, using FICE and WL in the right-sided colon including cecum, ascending colon, and transverse colon. The time needed for both insertion and examination for withdrawal and all lesions detected in the right-sided colon was recorded. Each patient was randomized in one of the following two groups with a computer-generated random number list; group A: after cecal insertion by WL, the colonoscope was withdrawn from the cecum to the splenic flexure with WL mode and then rewithdraw in the colonoscope with FICE from the cecum to the splenic flexure after reinsertion of the scope to the cecum by WL (WL-FICE); group B: withdrawing the colonoscope in the inverse order of group A (first FICE and then WL; FICE-WL). All lesions detected during either examination of FICE or WL were removed by endoscopic resection or biopsy specimens and sent for histological evaluation without exception. All lesions identified on the second examination were considered as lesions missed by the first examination. The location of each lesion was defined according to landmarks including hepatic flexure and splenic flexure. The size of the lesions was estimated using open endoscopic biopsy forceps.

### 2.4. Histopathological Evaluation

Resected specimens were immediately fixed in 10% buffered formalin solution and subsequently stained with hematoxylin-eosin. Experienced gastrointestinal pathologists who were completely blinded to each endoscopic diagnosis evaluated all pathological specimens. Histological diagnoses were determined according to the World Health Organization (WHO) criteria [14].

### 2.5. Statistical Analysis

This study was mainly designed to demonstrate that the colonoscope with FICE has a different reliability than with WL for polyp detection. No sample sizes were calculated, as this was a pilot study. The design of the study included two independent groups; group A underwent colonoscopy with FICE after colonoscopy with WL, and group B underwent colonoscopy with WL after colonoscopy with FICE. Categorical variables are expressed with frequencies and percentages. Continuous variables are expressed with means and standard deviations. Statistical differences were analyzed by *χ* ^2^ test of independence, the Mann-Whitney *U* tests, and Fisher's exact test. A *P* value of less than 0.05 was considered statistically significant. Statistical analysis was conducted with SPSS V. (Chicago, IL), Stat X act v. 5.0.3 (Cytel Co., MA), and Statistica v. 5.5 (Tulsa, OK).

## 3. Results

A total of 102 patients were enrolled in this study. Fifty-one were randomized to group A and B. According to the protocol, two cases were excluded from the final analysis because of impossible insertion cases to cecum bottom: one bowel adhesion case after operation in group A and one local recurrence of anastomosis in group B. A total of 100 cases were finally evaluated. The 100 patients included 61 (61%) men, and the mean age and standard deviation were 63 ± 12 years. The indications for colonoscopy were postoperative surveillance of anterior resection (*N* = 65) and sigmoidectomy (*N* = 35). The bowel preparation was described as excellent or good in 82 cases (82%) and fair in 18 (18%), respectively (Table 1).

There were no statistically significant differences between the FICE and WL with respect to withdrawal time, lesion detection, macroscopic finding, and tumor size. Total numbers of detected and removed lesions by FICE and WL colonoscopy were 65 and 45, respectively. Characteristics of the detected neoplastic lesions by FICE and WL colonoscopy were flat elevated: 53 (90%) and 33 (87%) and small (<5 mm): 33 (56%) and 24 (63%), respectively (Table 2).

The miss rate for all polyps with FICE (24%) was significantly less than that with WL (46%) (*P* = 0.03). Among all detected polyps, the number of neoplastic lesions detected by FICE and WL colonoscopy was 59 and 38, respectively. Among 45 neoplastic lesions, which were diagnosed in group B, 34 (76%) lesions were detected at the first FICE withdrawal technique (Table 3). In contrast, in group A (among 52 neoplastic lesions), only 27 (52%) lesions were recognized at the first WL withdrawal technique, and 25 (48%) lesions were detected by the second FICE examination. Significantly more neoplastic lesions were missed by WL compared with FICE system (*P* = 0.02).

## 4. Discussion

Detection of adenomas is essential at screening colonoscopy, however, the miss rate especially for small and flat lesions remains unacceptably high. According to several reports, 10 to 15% of lesions remains undiagnosed at colonoscopy, even by experienced practitioners. In this pilot study, we investigated the utility of a FICE system on miss rates during colonoscopy and the efficiency of colonoscopy withdrawal. Based on the results of our study, FICE system may be useful for the detection of colorectal adenomas in the right-sided colon compared to WL conventional colonoscopy under high-quality bowel preparation.

The largest advantage of this system may prove to be the ability to perform faster and more efficient examination without the need for additional attachments to the endoscope and without dye spraying or infusion. According to the National Polyp Study (NPS), the incidence of colorectal cancer was decreased by endoscopic intervention. In brief, polypectomy during routine colonoscopy has been shown to prevent the development of colorectal cancer, compared with the incidence of it in reference groups. Therefore, colonoscopy is considered as a gold standard for detection and treatment of colorectal adenomas, however, the conventional colonoscopy technique during withdrawal, even if very careful, cannot detect all lesions, especially flat and small depressed ones. Potential explanations for failure at colonoscopy include poor bowel preparation or inadequately short withdrawal times [15, 16]. Moreover, an important technical factor that determines the detection of lesions is the level of mucosal contrast provided by the imaging method. Low contrast might contribute to the miss rate of small and flat lesions that show only subtle changes in mucosal topography, focal pallor, and marginal irregularity [17, 18].

Endoscopic imaging techniques aimed at early detection of colorectal cancer and its precursors have been developed over the last decade. Techniques that improve the detection of mucosal irregularities, such as pancolonic chromoendoscopy, narrow band imaging (NBI), high-resolution imaging, autofluorescence imaging, and FICE have been applied in a variety of clinical situations to enhance the detection of flat and depressed lesions or to enable histological diagnosis. Many authors have reported that chromoendoscopy is helpful for the detection and detailed morphological assessment of flat and depressed colorectal lesions [19–28]. Pancolonic chromoendoscopy using an indigocarmine diffusion during withdrawal from the cecum, which highlighted subtle mucosal irregularities, has been reported to significantly increase the detection of diminutive, flat neoplastic lesions in the right colon. However, the withdrawal time for the indigocarmine dye spray group was almost twice as long as for the control group.

Computed virtual chromoendoscopy with FICE is a novel optical approach to enhance mucosal contrast [29]. This technique enhances the bandwidth of light components, resulting in dye-less contrast enhancement of mucosal and vascular details. To overcome the problems of conventional chromoendoscopy, another chromoendoscopic techniques FICE and NBI were recently developed. Both techniques are safe, rapid, and easy to apply, and several preliminary studies reported enhancement of vascular and mucosal contrast. The NBI system has been shown to be helpful in visualizing such lesions by improving contrast and is considered to be a new type of optical/digital chromoendoscopy [30, 31]. In particular, magnification using NBI colonoscopy for the observation of the presence of “meshed brown capillary vessels” is extremely useful for distinguishing between neoplastic and nonneoplastic lesions without any dye solution. Regarding polyp detection, however, it is controversial at this moment [32]. Furthermore, during NBI colonoscopy examinations, intestinal fluid was seen as being reddish in color similar to blood. Therefore, proper bowel preparation is one of the limitations when using this system.

In 1989, Miyake et al. [9] developed and reported a new optimal band imaging system, and endoscopic examinations with this optimal band imaging system were developed as FICE after these essential reports. Images acquired by this new system provided better brightness than old fiberscopic images. Preliminary reports showed that in the esophagus, the detection rate for neoplasm of FICE and NBI appears similar to that of conventional chromoendoscopy [33, 34]. In other reports, FICE with high-definition endoscope in colonoscopy or upper GI endoscopy was useful for diagnosis between neoplastic and nonneoplastic lesions [35–37]. Pohl et al. reported that FICE was superior to standard colonoscopy and equivalent to conventional chromoendoscopy for distinguishing neoplastic from nonneoplastic lesions and adenoma detection rate was not improved by FICE compared to WL with targeted indigocarmine spraying [38, 39]. However, there are few prospective studies that have attempted to clarify the usefulness of the adenoma detection rate using FICE system [40].

In this study, a total of 110 lesions from 100 patients were detected and removed endoscopically. Among these lesions, the number of neoplastic lesion detected by FICE and WL was 59 (91%) and 38 (84%), respectively. In contrast, the number of nonneoplastic lesions recognized as a polyp and removed by FICE and WL colonoscopy was only 6 (9%) and 7 (16%), respectively. The lesions we diagnosed and resected in this study with FICE and WL systems were mostly neoplastic ones. However, we consider further investigation is necessary to evaluate the efficiency for differential diagnosis with FICE system. Diminutive flat elevated lesions are thought to be of little clinical significance because such lesions, especially less than 5 mm polyps, are low-grade dysplasia in most cases. Meanwhile, depressed lesions are considered to have a high malignant potential compared to polypoid ones in similar size [41–43]. In this present study, all detected lesions' macroscopic type was flat elevated or polypoid. Because of low incidence, there were no depressed lesions in this study. However, significantly more small and/or flat neoplastic lesions were detected by FICE compared with WL colonoscopy. Additionally, the brightness of the image during FICE colonoscopy is sufficient to ensure a good overview in large luminal diameter sections of the bowel. Therefore, FICE colonoscopy is considered to be a promising modality to detect small depressed lesions.

Bowel preparation rate of excellent or good in our study was described more than 80 percent in both group. Negative advocacy piece to improvement in detectability of colorectal polyps using FICE was described in the past report with lower bowel preparation rate of excellent or good less than 75 percent [44]. It is suggested that proper bowel preparation is indispensable to achieve success to detect small colorectal lesions, so we think quality of bowel preparation is very important for full effectiveness of FICE colonoscopy.

There are several limitations in our study. First, this study was performed at a single institute as a pilot study. Our data are precise but it is uncertain whether it would be available for all examiners. Therefore, additional multicenter studies are necessary to clarify the usefulness of FICE system.

Another point worth mentioning is that our study was conducted within the limits of the right colon, which mean withdrawal time were more than three minutes. We selected modified back-to-back colonoscopy in right-side colon. Complete back-to-back colonoscopy may be painful for patients under no sedation and longer procedures because many colonoscopies without sedation are usually performed in Japan. The higher prevalence of flat and diminutive lesions diagnosed in the right colon may be consistent with past descriptions [45, 46]. Furthermore, a higher miss rate of detection has been reported in the right colon compared to the left colon. Therefore, we defined the area from the cecum to the splenic flexure as the target area in our prospective study. We think that it is necessary to evaluate the total colonoscopy using FICE from cecum to rectum as further estimation.

In conclusion, colonoscopy using FICE could beneficially enhance the detection of neoplastic lesions in the right-sided colon, especially flat and/or diminutive adenomatous lesions compared to conventional WL colonoscopy under proper bowel preparation.

## Figures and Tables

**Table 1:** Patient characteristics.

	Group A (WL-FICE)	Group B (FICE-WL)
Cases	50	50
Male	30	31
Female	20	19
Mean age (yr)	62.7	63.3
Operation history		
Anterior resection	36	29
Sigmoidectomy	14	21
Bowel preparation		
Excellent	23	17
Good	19	23
Fair	8	10

**Table 2:** Comparison FICE with white light.

	FICE	WL
Withdrawal time (sec.)	213	193
(Range)	(90–490)	(79–600)
Detected lesions		
All	65	45
Neoplastic	59 (91%)	38 (84%)
Macroscopic finding		
Flat elevated	53 (90%)	33 (87%)
Polypoid	6 (10%)	5 (13%)
Tumor size		
<5 mm	33 (56%)	24 (63%)
≥5 mm	26 (44%)	14 (37%)

**Table 3:** Detected lesions in group A and B.

	A (WL-FICE) (*n* = 50)	B (FICE-WL) (*n* = 50)	*P* value
Total number of lesions (%)			
First	WL 33 (54)	FICE 37 (76)	*P* = 0.03
Second	FICE 28 (46)	WL 12 (24)	
Total number of neoplastic lesions (%)			
First	WL 27 (52)	FICE 34 (76)	*P* = 0.02
Second	FICE 25 (48)	WL 11 (24)	
